# Study of Data Transfer in a Heterogeneous LoRa-Satellite Network for the Internet of Remote Things

**DOI:** 10.3390/s19153384

**Published:** 2019-08-01

**Authors:** Ivan Lysogor, Leonid Voskov, Alexey Rolich, Sergey Efremov

**Affiliations:** 1 Laboratory of the Internet of Things and Cyber-physical Systems, National Research University Higher School of Economics, Moscow 101000, Russia; 2 Department of Computer Engineering, National Research University Higher School of Economics, Moscow 101000, Russia; 3 School of Business Informatics, National Research University Higher School of Economics, Moscow 101000, Russia

**Keywords:** internet of remote things, heterogeneous networks, terrestrial-satellite networks, SATCOM, Iridium SBD, LoRa, Protobuf

## Abstract

In the absence of traditional communication infrastructures, the choice of available technologies for building data collection and control systems in remote areas is very limited. This paper reviews and analyzes protocols and technologies for transferring Internet of Things (IoT) data and presents an architecture for a hybrid IoT-satellite network, which includes a long range (LoRa) low power wide area network (LPWAN) terrestrial network for data collection and an Iridium satellite system for backhaul connectivity. Simulation modelling, together with a specialized experimental stand, allowed us to study the applicability of different methods of information presentation for the case of transmitting IoT data over low-speed satellite communication channels. We proposed a data encoding and packaging scheme called GDEP (Gateway Data Encoding and Packaging). It is based on the combination of data format conversion at the connection points of a heterogeneous network and message packaging. GDEP enabled the reduction of the number of utilized Short Burst Data (SBD) containers and the overall transmitted data size by almost five times.

## 1. Introduction

The task of deploying data collection networks in regions without any existing infrastructure poses a number of challenges for engineers. With the Internet of things (IoT) spreading its influence in cities and their suburbs, it is still an open question of how IoT can further extend in regions such as the Arctic or Antarctic, unpopulated deserts or tropical forests. The concept of smart autonomous devices operating in remote undeveloped areas has recently gained its own name—the Internet of Remote Things (IoRT) [1].

There are a number of IoRT application areas, in which satellite communication (SATCOM) is of primary importance, being the only feasible option of integrating a local data collection system into the global network—shipping, vessel tracking, marine engineering [2], smart grid [3,4], ecological monitoring, emergency management and medicine [4], earthquakes, flash floods, terrorist attacks and tsunami detection [5,6], oil and gas industries [7] and backhaul connectivity [3,8]. Other notable scenarios include the Internet of Underground Things (IoUT) [9], the Internet of Cultural Things (IoCT) [10,11], the Internet of Arctic Things (IoAT) [12,13] and security and military affairs (the Internet of Battle Things, IoBT) [14,15]. In general, the organization of ubiquitous internet access using backhaul space communications is discussed in a wide range of research papers [16,17,18,19,20,21,22,23,24,25,26,27,28,29,30,31].

Apart from the obvious technical difficulties related to placing sensors in an unsupervised aggressive environment, the key problems in satellite IoRT are the high cost of sending messages and the occurrence of collisions and significant delays [4,13,15,17,19,23,24] in data acquisition and transmission. As a result, plenty of algorithmic issues are still being researched, with authors presenting new methods of channel access [27,28,29], modulation techniques [2,25], routing mechanisms [17,23,29], load balancing [23,29], data transfer algorithms and protocols [6,20,26,28] and methods of increasing network throughput [19,31], as well as schemes of reducing power consumption of the network infrastructure [19] and lowering the cost of message transmission [23]. In addition, new architectures [12,21,22,30,31] of heterogeneous IoT-satellite networks, including the ones based on long range (LoRa) [18,25,32] and Iridium [3,13,15,17,33] have been proposed.

Energy efficiency remains the main requirement for data transmission in the Internet of things as it directly affects the lifetime of autonomous devices. One of the factors that influence energy efficiency is the amount of transmitted data or the ratio between service data and useful payload. Optimizing such a ratio can be done by changing the existing network algorithms and protocols. In a heterogeneous structure, novel algorithms and approaches can be applied on the edge of the two networks, which is done in our work and which we find as the main distinctive feature compared with other existing approaches.

We propose an architecture of a hybrid IoT-terrestrial-satellite network and a two-step Gateway Data Encoding and Packaging (GDEP) method for the LoRa-Iridium configuration that transforms a sequence of incoming LoRa Message Queuing Telemetry Transport (MQTT) messages into an output sequence of Protocol Buffers (Protobuf) Short Burst Data (SBD) containers.

We present our results in the following order: Section 2 contains an overview of related works. Because of the vast array of technologies existing both on the terrestrial side and the satellite side, in Section 3 we first justify our choice of standards and protocols, then present an architecture of a low power wide area network (LPWAN) [34]-satellite network for the Internet of things in hard-to-reach areas. Further on, we describe our encoding and packaging method. In Section 4, we review results of experimental verification of the main hypotheses using semi-natural simulation.

## 2. Literature Review

De Sanctis et al. [1] provide a comprehensive overview of IoRT with SATCOM focusing on the architectural patterns of interconnection between satellite and sensors/actuators. Three typical applications, in which satellites could play an important role, are outlined: smart grid, environmental monitoring and emergency management. In terms of the satellite standards and protocols, the authors consider digital video broadcasting – return channel via satellite (DVB-RCS2) as the main feasible option that can guarantee a certain Quality of Service (QoS) level, only briefly mentioning low Earth orbit (LEO) constellations.

In [4], the authors study the applicability of satellite communication for time-critical IoT applications, such as in smart grid or e-health services. It is concluded that most of today’s applications have delay tolerances that are too stringent to be met by satellite links. However, there are certain types of applications that may still benefit from using SATCOM. A similar issue is addressed by Qu et al. [17]. In their research paper, the authors divide the possible IoT application scenarios into two groups: Delay-Tolerant Applications (DTA) and Delay-Sensitive Applications (DSA). It is argued that Low Earth Orbit satellite constellations in many cases offer more advantages compared with Geostationary Earth Orbit (GEO) systems. They also discuss a number of questions related to system design of terrestrial IoT and satellite networks, including constellation design, network architecture and access and routing protocols.

Palma and Birkeland [12] propose an Internet of Arctic Things architecture, which is characterized by a freely drifting swarm of small satellites and satellite-aware routing as one of the advanced options. The researchers conducted an experimental assessment with several ground nodes and satellite orbits, as well as lightweight communication protocols, which resulted in a low overall number (<5%) of packet losses.

In terms of the engineering design idea, [3] provides the closest setup to the prototype discussed in the present paper. The work describes an IoT system design based on ZigBee that uses the Iridium satellite constellation for remote monitoring and control purposes in remote Himalayan villages.

The research of Bacco et al. [28] focuses on the comparison between two IoT/Machine to Machine (M2M) protocol stacks in relation to data transfer over a random access satellite channel based on the DVB-RCS2 standard. The simulation showed that the Constrained Application Protocol (CoAP) outperforms MQTT on low and moderate traffic rates.

In summary, all of the aforementioned works study the feasibility of satellite communication for IoT, formulating many open issues and addressing some of them. We see a potential gap in data transfer optimization on the edge of the two network parts—the terrestrial part and the satellite part. That is the primary aim of our study.

## 3. Heterogeneous Network Architecture

### 3.1. Conceptual IoT-Satellite Network Architecture

**Figure 1:**
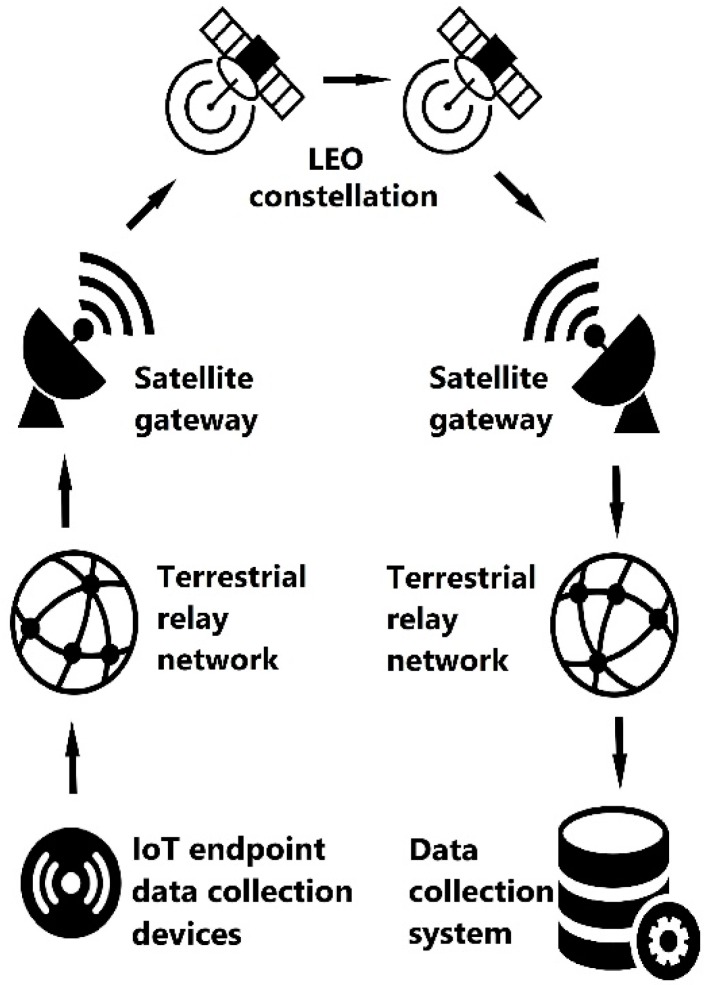
Conceptual Internet of things (IoT)-satellite network architecture.

A conceptual architecture of an IoT-terrestrial-satellite network that will be taken as the basis for our further discussion is shown in Figure 1. A set of remote endpoint devices collect information and pass it through a terrestrial relay network to a satellite gateway, which then forwards it through a LEO constellation to a receiving station and then further to a data collection system.

The scalability, reliability and energy efficiency of the network all depend on the choice of data transfer technologies and underlying protocols. Such choice also defines constraints on the amount of information transmitted in a single message and the format of transmitted messages. Currently, there are many technologies and protocols that differ in communication range, speed, frequency bands and modulation used during data transmission.

### 3.2. Long-Range Technology for Hybrid Terrestrial-Satellite Networks

When choosing data transfer technologies and protocols in the heterogeneous IoRT setup, we consider the following key criteria:

- energy efficiency;
- area coverage from a single base station;
- cost of service;
- possibility of using unlicensed radio frequency ranges.

Low Power Wide Area Networks (LPWANs) [34] are usually considered as the best last mile technology for the Internet of Remote Things. The main distinctive features of LPWANs are:

- long data transmission distance. In many implementations it can reach 10 km or even more;
- higher energy efficiency compared with standard data transfer protocols.

The limitations of the technology are low speed and possible significant delays in data transmission. One of the commonly used LPWAN specifications is LoRa [32], with the Long Range Wide Area Network (LoRaWAN) [32] protocol being based on it. LoRa uses linear frequency modulation in the unlicensed frequency range up to 1 GHz (the exact range depends on the country). An adaptive spread spectrum technology allows one to dynamically change the bandwidth depending on the range of frequencies used. Thus, the LoRa specification allows for maximum transmission distance and energy efficiency compared with many other data transfer technologies for the Internet of things. The LoRaWAN protocol defines the primary network architecture for LoRa, in which a network gateway plays the role of a transparent bridge, passing LoRa packets onto the network and vice versa. LoRa and LoRaWAN are very often considered as a single joint technology covering several network layers.

An alternative technology used in transmission of IoT data is Narrowband-IoT (NB-IoT) [35]. The technology was developed by the 3rd generation partnership project (3GPP) consortium and is now integrated into the long-term evolution (LTE) standard [36]. It is a subset of LTE to be used in energy-efficient stand-alone devices. Compared with LTE, the NB-IoT technology does not support roaming between base stations and channel aggregation to improve performance. In addition, it requires cellular communications and cannot be used in hard-to-reach areas. As a result, we will not consider NB-IoT as an equal alternative, and will focus on LoRaWAN as the primary technology for sensor data acquisition.

With respect to satellite backhaul, there are two main providers of digital data transmission as of today: Inmarsat [37] and Iridium [33].

Inmarsat satellites belong to the class of GEO systems, providing coverage up to 70° north and south latitude, that is, they reach almost the entire globe except for “snow caps”. The satellite system offers an Internet connection at speeds of up to 384 Kbps. A key feature of the platform is the presence of a controller, which allows implementing applications in a short time. There are ready-made Machine to Machine (M2M) [38] solutions, the choice of which depends on the complexity, speed and volume of the transmitted and received information. Among such solutions are IsatM2M [39], IsatDataPro [40] and BGAN M2M [41].

The Iridium satellite system, in turn, is a LEO constellation that provides data rates of up to 128 Kbps. The operator company offers M2M equipment based on Short Burst Data (SBD) 9602, 9603N and 9523 satellite modems, which allow transmitting short data messages. The key features of SBD satellite modems are their low power consumption and text messaging capabilities.

The requirement to provide communication services in remote areas leads to the need for a coverage zone in the regions of the Far North and the Arctic. The only SATCOM that meets this criterion is the Iridium satellite system. Connecting an SBD Iridium modem to each IoT sensor is not feasible both in terms of cost and energy consumption. Thus, to organize the connection of IoT sensors via satellite communication channels, it is necessary to provide a gateway between the LPWAN network and the satellite communication channels. This design aligns with our conceptual model of Figure 1 and will be further detailed in Section 3.4

### 3.3. Data Formats for Hybrid LoRa-Satellite Networks

During information transmission through a terrestrial network and further through a LEO satellite constellation, the method of information encoding is of great importance, since it affects the ratio of application and service data in the overall information stream. In our work, we focus entirely on the upper OSI layers, leaving the transport part of the stack unchanged.

During the transfer of information between a single IoT sensor and the LoRaWAN gateway, only sensor data with a minimal payload can be transmitted. In general, an IoT device can be used to transmit readings of more than one sensor. In this case, each sensor’s value is complimented with an identifier or parameter name. When further transmitting information from the LoRaWAN gateway to the central data collection and analysis system, the end device identifier and the network parameters should be added to the data. As a result, most IoT systems use a key-value format for information encoding. With the modern trend of using open standards, we will omit some of the proprietary binary solutions and instead discuss the following open specifications designed for M2M connectivity: JavaScript Object Notation (JSON), binary JavaScript Object Notation (BSON), Concise Binary Object Representation (CBOR), MsgPack, JSONC and Protobuf.

The JavaScript Object Notation (JSON) format [42] is a subtype of the Abstract Syntax Notation 1 (ASN.1) format and is currently the de facto standard for transmitting information in web applications, as well as IoT systems. The textual representation of information in JSON makes it possible to analyze the obtained values without decoding, and the transmission of information in the “key-value” format increases flexibility. The availability of libraries for processing JSON messages in most programming languages makes application development much easier. At the same time, the textual basis of the format leads to larger message sizes. When each message contains all field definitions, the increase in the message size can be very significant.

The binary JavaScript Object Notation (BSON) format [43] is a binary version of JSON, which allows transmitting binary data in messages instead of text. Depending on the type of encoded information, it may be more efficient than JSON, but generally corresponds to it in terms of the message size.

The Concise Binary Object Representation (CBOR, RFC7049) [44] and MsgPack [45] formats use binary data representation and reduce the amount of data transferred by changing the format of the values transferred. These formats do not require the use of text field delimiters; they convert textual representations of integers, floating-point numbers and dates into a binary representation, which reduces the number of transmitted messages. Both the CBOR format and the MsgPack format do not require a preliminary definition of the transmitted data fields and are comparable in terms of the ratio of useful data to the total amount of transmitted data. The JSONC format [46] assumes the use of data compression using the zlib algorithm (RFC1950) without analyzing the transmitted data, and the degree of compression depends on the size and nature of the transmitted data. When transmitting information from IoT sensors, the message data structure can be defined in advance and, thus, field names may be omitted from each transmitted message.

Structured information can also be transmitted using the Protocol Buffers (Protobuf) format [47]. Protobuf is a highly specialized binary format, in which the reduction in the size of the transmitted data is achieved through the optimal representation of data types in binary form and the use of predefined message structures. Thus, in key-value pairs, only the binary key identifier is transmitted, and not its name, which significantly reduces the amount of data transferred.

The summary of data presentation formats with their key properties is presented in Table 1.

Considering the predetermined nature of data structures transmitted from IoT sensors, Protobuf seems to be the best choice, as it minimizes the amount of service information while still preserving the key-value approach. The key technical task, however, is to seamlessly integrate the protocol into an existing network infrastructure.

### 3.4. Heterogeneous LoRa-Iridium Network Architecture

**Figure 2:**
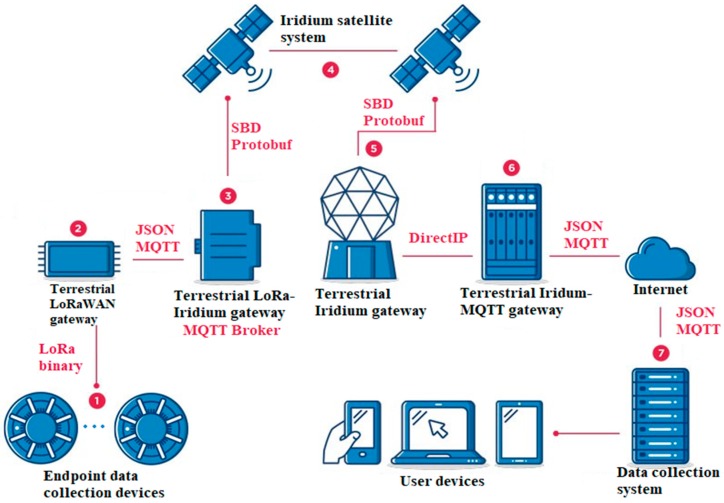
Heterogeneous long range (LoRa)-Iridium network architecture.

We considered a network architecture (Figure 2) to study the applicability of the proposed GDEP method. Elements of the architecture included:

1. Endpoint data collection devices. One or several sensors can be connected to these devices; data transmission is performed according to the LoRa specification.
2. Terrestrial LoRaWAN gateway.
3. Terrestrial LoRa-Iridium gateway. MQTT broker is co-located with this element.
4. Iridium satellite system for hybrid terrestrial-satellite network organization.
5. Terrestrial Iridium gateway.
6. Terrestrial Iridium-MQTT gateway.
7. General data collection and analysis system.

We performed the task decomposition and defined the main stages of information transfer from endpoint data collection devices (1) to the data collection and analysis system (7).

Each of the endpoint data collection devices (1) can have one or multiple sensors (e.g., temperature, vibration, light), and transmit data in the LoRa binary format to the LoRaWAN gateway (2). The LoRaWAN gateway (2) acts as a transparent bridge passing the information to a connected MQTT service through a broker. This information is encoded in the JSON format. The number of published MQTT messages corresponds to the number of received packets on the LoRa transmission channel. The LoRa-Iridium gateway (3) retrieves messages from the LoRaWAN gateway (2) through an MQTT broker. We assumed there were no constraints on the channel width and on the energy efficiency of transmission between (2) and (3) as they were equipped with a stationary power supply and communicated with each other via a high-speed connection.

We selected MQTT as one of the standard and widely used protocols for data transfer in the IoT. The protocol was initially developed at IBM [48] and Arcom (now Cirrus Link) for economic data transfer from oil pipelines via a satellite channel. In 2010, protocol specifications were published, and in the fall of 2014, MQTT version 3.1.1 became an open standard for the Organization for the Advancement of Structured Information Standards (OASIS). Two papers [49,50] provide comparisons of session layer protocols outlining the efficiency of MQTT for IoT.

The main task of the LoRa-Iridium gateway (3) is to convert messages coming from the terrestrial IoT network into the appropriate format of the SBD service.

Each SBD message is transmitted via the satellite constellation (4) and the Iridium terrestrial gateway (5) to the Iridium MQTT gateway (6), which decodes messages back from SBD to JSON and publishes them through another MQTT broker to the data collection system (7).

## 4. Gateway Encoding and Packaging Method (GDEP)

**Figure 3:**
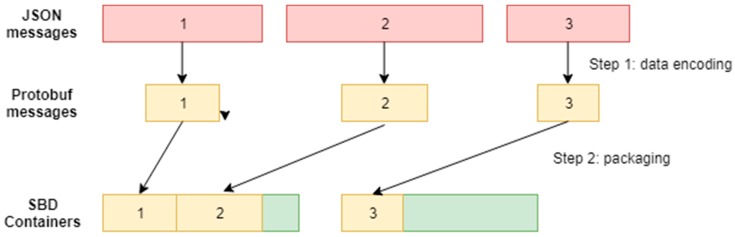
Scheme of the Gateway Encoding and Packaging (GDEP) method.

The GDEP method aims to optimize the amount of data transferred through a satellite link, which is the architectural bottleneck both in terms of speed and cost. The method consists of two connected steps (Figure 3). At the first step, a format conversion is performed resulting in reduction of overall data size, and at the second step, the packaging algorithm is additionally applied, which leads to a higher utilization rate of the satellite channel.

Step 1. Data encoding

To evaluate the effectiveness of the first step, we performed a network simulation taking into account the following constraints associated with network standards, protocols and hardware:

- The maximum payload size of the LoRaWAN message is 222 bytes when sending messages through an intermediate device and 242 bytes when sending information directly between the endpoint device and the gateway [51];
- The maximum SBD payload size is 340 bytes [52,53];
- The raw transmission delay in the LoRa network can vary from 60 to 1250 ms.

The technical details of how JSON messages were encoded in Protobuf can be found on our GitHub project page [54].

One thousand LoRa messages from endpoint devices with different amounts of sensors—from 1 to 13—were generated, then we compared the amount of transmitted data using the JSON and Protobuf formats. The generation of events by endpoint devices was carried out independently of each other with a fixed average probability value of an event occurring, so we used the Poisson distribution to determine the number of messages from the endpoint devices in one message from the sensor [55]. We assumed that the values of the endpoint devices would obey the normal distribution law. The simulation results are shown in Table 2 below.

Format conversion reduces the size of transmitted data by an average of 4.8 times. When using the Google Protobuf data presentation format, the size of the resulting IoT message on the terrestrial network varies from 70 to 126 bytes depending on the number of sensors (from 1 to 13). The maximum size of the SBD transmission is determined by the hardware, and in our case is 320 bytes; thus, it becomes possible to pack several Internet of things messages in one SBD transmission session. Upon receipt of the incoming messages, the LoRa-Iridium gateway buffers them and, when the buffer is larger than a specified size, packages the messages to perform transmission using the Iridium SBD communication channel.

Step 2. Message packaging

The packaging step can be defined as a standard optimization problem. Let us define the following variables:

n—the number of types of sensor messages in the buffer;
m—the number of SBD containers;
$b_{j}$—the number of messages of type j, $j=\bar{1..n}$;
$v_{j}$—the volume of messages of type j (in bytes), $j=\bar{1..n}$;
$x_{ij}$—the number of messages of the j-th type in the i-th SBD container, $i=\bar{1..m,\;}j=\bar{1..n}$;
$y_{i}$—flag indicating whether a container is empty (0) or not (1);
S—size of the SBD container (in bytes)

(1) $$\min\sum_{i=1}^{m}y_{i}.$$

Subject to the following constraints:

The total size of messages fitted into one container cannot exceed the corresponding container size

(2) $$v_{i}x_{i1}+\ldots +v_{n}x_{in}\leq S,\;i=1..m.$$

Since all messages of each type have to be transmitted,

(3) $$x_{1j}+x_{2j}+\ldots +x_{mj}=b_{j},\;j=1..n\;.$$

We define the lower limit of the number of messages $m^{\prime }$—the number of transmitted packets, for which buffer messages from the buffer with the volume V can be transmitted with the volume of the short message S, subject to the constraints (2) and (3):

(4) $$m^{\prime }=\frac{\sum_{j=1}^{n}v_{j}\cdot b_{j}}{S}.$$

Additional constraints can be defined in simulation modelling. These can be the lifetime of a single IoT device, the rate at which new messages arrive and, as a result, the number of simultaneously open SBD containers.

The total transmission time of each message from endpoint devices to the data collection system should not exceed the threshold value—T_p_. The main task will be to choose the most suitable algorithm that uses the smallest number of containers and introduces a delay in the process of sending messages no more than T_p_.

Since the packing problem is NP-complete, the minimum number of required containers can be determined only by the exhaustive search method. We consider several existing approximate packaging algorithms [56,57]:

Algorithm 1: “First suitable with one open container”. Messages are selected sequentially from the buffer and placed in the first container. If a new message cannot be placed in the current container, then a new container is opened. The filled container is closed and shipped.

Algorithm 2: “First suitable with multiple open containers”. Messages are selected sequentially from the buffer and placed in the first container. If a new message cannot be placed in the current container, then the next free container is used. Unlike the previous algorithm, all containers remain open and are closed only after all messages from the buffer are placed.

Algorithm 3: “First suitable with multiple open containers and message sorting”. The messages in the buffer are sorted in descending order of size. After that, messages are selected sequentially from the buffer and placed in the first container that has sufficient space. All containers remain open and close only after all messages from the buffer are placed.

We compared the operation of the three algorithms using the architecture of a heterogeneous network (Figure 2). One thousand LoRa messages were generated from sensor nodes with different amounts of data—from 1 to 13 (the number of messages is determined by the Poisson distribution)—and messages were packed using the three algorithms under consideration. The simulation results are presented in Table 3.

It can be concluded that Algorithm 1 is no more than 2% worse than algorithm 3. The difference in contained utilization between algorithms 1 and 3 is less than 1%. Given the limitations on the delay in sending messages and the resource-intensiveness of the algorithm, such a difference can be neglected.

The delay associated with the process of packing depends on the number of open containers and is equal to the number of messages in one container multiplied by the number of open containers. Thus, to reduce the delay, we can only use algorithms with a minimum number of open containers.

## 5. Experimental Results

**Figure 4:**
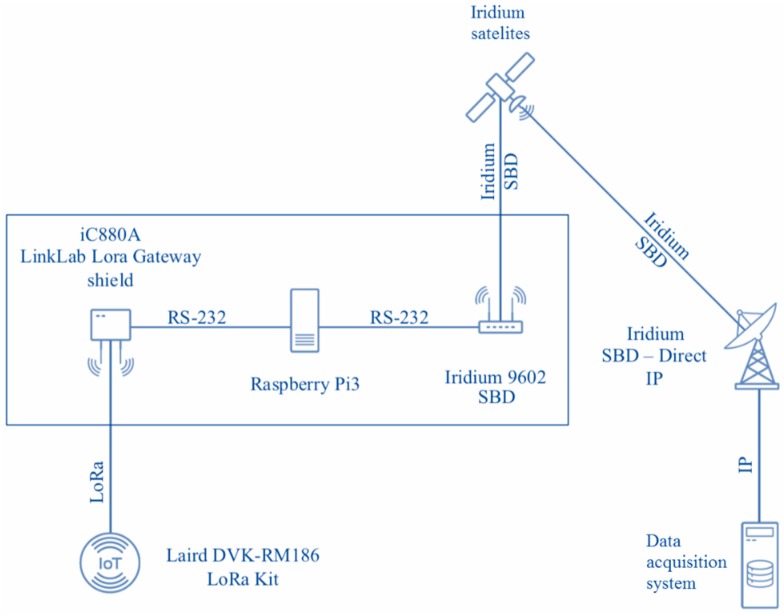
Scheme of the experimental stand.

To verify the obtained results, an experimental stand consisting of the Laird DVK-RM186 kit, the LinkLab LoRaWAN Gateway shield, a Raspberry Pi3-based IoT MQTT station, the Iridium 9602 SBD modem and DirectIP (SBD) and MQTT servers was built (Figure 4). The latter two components were installed on a virtual cloud server. For message packing, algorithm 1 was used.

During the experiment the stand components interacted as follows:

1. An end device from the Laird DVK-RM186 kit generated LoRa messages with a variable inter-arrival time from 1 to 20 s and a payload from 18 to 20 bytes. The messages themselves consisted of a pseudo-random sequence of characters emulating sensor data.

2. Messages were transferred through the LoRaWAN gateway and persisted at a local MQTT server installed on the Raspberry PI based IoT station [58].

3. Messages were then retrieved from the MQTT server into a First-In First-Out (FIFO) queue with the format converted from JSON to Protobuf.

4. In the first scenario, each new message from the queue was placed in a new SBD container and immediately passed to the Iridium satellite modem. In the second scenario, a packaging algorithm was additionally applied.

5. The Iridium 9602 modem normally responded with a confirmation within 5 s, indicating either successful transmission or failure. If the message transmission failed, an attempt was repeated up to 10 times. After 10 transmission failures, the message was deleted from the queue.

6. After sending the message, the main characteristics related to the data transfer process—send status, size of the initial JSON message, size of the encoded Protobuf message, total transfer time—were recorded on the IoT station side, as well as on the server side.

7. Steps 4–6 were repeated for subsequent messages from the queue.

**Figure 5:**
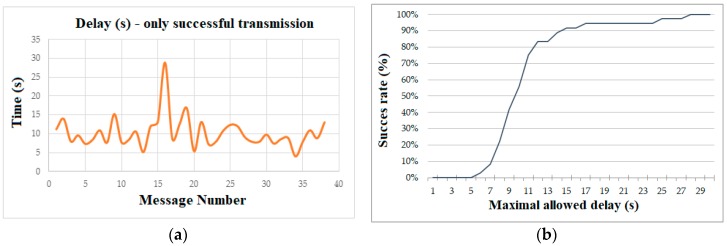
SBD message transmission through the Iridium network: (**a**) raw delay data; (**b**) success rate as a function of maximal application delay.

The experiment was carried out in an open area in the Moscow region. The data transfer process within each experimental setup lasted for about 12 min, then the LoRa message generation frequency was increased and the experiment was repeated. Within the 12-minute interval, about 40 SBD containers were sent, with 95% of them successfully arriving at the server side. The message transmission time related to passing data onto the Iridium network varied from 6 to 27 s (see Figure 5a), averaging at 10.7 s.

These data can be interpreted from a different perspective, as shown in Figure 5b. As mentioned in the introduction, significant delays in the data transmission process are one of the main limitations of satellite technologies. With most real-life applications having certain Quality of Service (QoS) requirements to the maximal possible delay of information transmitted through the underlying network, the feasibility of the satellite channel can be determined by figuring the transmission success rate. Transmission success is determined by a message received at the server endpoint within a given interval.

**Figure 6:**
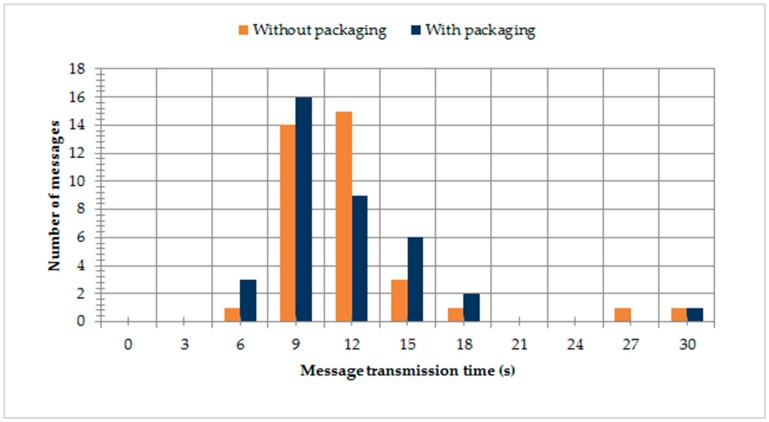
Distribution of delivery time through for the frequency of 1 message per second.

**Figure 7:**
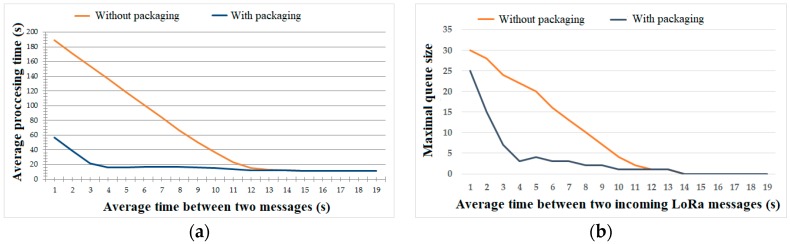
Satellite network performance as a function of inter-arrival time of LoRa messages: (**a**) average message processing time (s); (**b**) maximal queue size.

Figure 6 shows the distribution of delivery time with and without the packaging algorithm applied. As we expected, there was no significant difference in the two scenarios, as packaging only affected the total size of each container, which had a small influence on the network delay. However, measuring the Iridium latency on its own was not our primary aim. The most important characteristic in our experimental setup was the full processing time of each message from the moment it arrived from the LoRa network (Figure 7a). Here, the effect of the packaging algorithm can be seen much more clearly. Without packaging applied, new messages had to wait for the previous ones to be processed by the Iridium network. A container packaging algorithm enabled a steady network operation already at the rate of 1 LoRa message in 4 s.

The second key characteristic was the maximal queue size formed of incoming LoRa messages (Figure 7b). It was quite obvious that when the satellite network was not able to process all incoming messages in time, the queue size grew infinitely unless messages were discarded. This directly correlates with the previous result related to the average message processing time. The queue size went down to 0 as soon as each incoming message was immediately processed by the Iridium modem.

**Figure 8:**
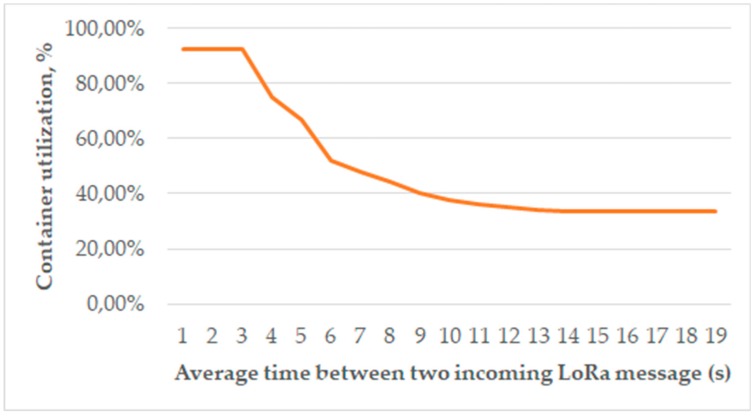
Container utilization with packaging applied.

A packaging algorithm can be evaluated by the container utilization rate. In our work, we studied the case of a fixed container size, which on average accumulated three incoming LoRa messages. As shown in Figure 8, packaging was extremely important for high-load network scenarios, with the container utilization rate reaching above 90%. When the rate of incoming messages fell below 0.1 messages/second, all containers accumulated no more than a single LoRa frame; hence, the utilization rate stabilized at roughly 33%, which matches simulation results (Table 2, row 2).

## 6. Discussion

In this paper, we have proposed an architecture of a hybrid LPWAN-satellite communication network that takes into account the present state-of-the-art of the Internet of things with its standards and protocols, and makes it possible to deploy IoT systems in remote areas. Focusing on data transfer optimization, we proposed a data format change on the presentation layer that can be implemented in the gateway supplemented with a packaging algorithm.

Our experimental results related to the Iridium network align well with the previous findings of other authors [3,13,15,17]. The average latency in SBD mobile-originated (MO) message transmission is about 10 s. Such a value is certainly significant enough to limit the application scenarios to the ones which deal with delay-tolerant data only. In this sense, the case of the Internet of things with sensors measuring environmental parameters that change slowly, this seems to be a perfect application area.

With sensors deployed in an area without any existing network infrastructure, the LoRa technology seems to be one of the best choices currently available. The task of aggregating data from IoT sensors and transmitting them to the global network over satellite involves many levels of optimizations. In the present paper, we only discuss those which happen on the edge of the two networks: LPWAN and satellite.

Optimizing data transfer enables the ability to either increase the number of sensors using the same gateway or to lower their power consumption, which is extremely important for scenarios of autonomous battery-powered devices or devices that use alternative energy sources.

It has to be mentioned that using satellite communication has very significant limitations—high cost of service, low throughput and long delays. However, we still believe it to be the most feasible option when building a data acquisition system in remote regions without any terrestrial communication infrastructure.

## 7. Conclusions

As a result of this work, an architecture of an IoT technological stack in hard-to-reach areas was presented. Information encoding methods were identified that allow for the ability to solve the problem of collecting information from remote sensors located in the absence of traditional communication channels, and practical verification of received results was obtained. The paper used simulation modelling to study the applicability of different methods of presenting information in the case of transmitting IoT data over low-speed satellite communications channels.

We see the following main contributions of this paper:

1. A novel architecture of a heterogeneous data collection network for remote areas of the world, which includes energy-efficient technologies of the Internet of things at the data collection level, with low-speed satellite communication channels targeted at M2M and IoT being proposed.
2. A new method (GDEP) of encoding information at the OSI presentation layer of a heterogeneous IoT network was developed, which by combining several encoding and packaging techniques, increases the efficiency of data transmission by 4.8 times.
3. The proposed GDEP method was validated on a simulation model and on experimental equipment.

The GDEP method proposed in the paper allows for the use of IoT technology stack in remote regions by integrating it with the SBD satellite short message service. The GDEP method allowed for the reduction in the volume and number of SBD messages during data transmission via low-speed satellite communication channels, which made it possible to reduce the size of transmitted data by almost five times. Reducing the cost of data transmission leads to an increase in the economic efficiency of SATCOM for organizing data transmission networks in remote areas, which do not have a telecommunication infrastructure.

## 8. Future Works

The Internet of remote things (IoRT) is a recently introduced paradigm describing monitoring and control networks in hard-to-reach areas. These networks usually have very limited throughput and tend to be heterogeneous, which opens a way to new models, methods and algorithms for optimizing data transfer, energy efficiency and traffic balancing. We have identified several tasks that need to be addressed within the framework of IoRT and that can benefit from the findings of the present paper.

We believe that the GDEP method can be further improved and generalized for the satellite-IoT scenario with any combination of initial data transfer technologies, encoding formats and container sizes. This will require additional simulation and experimental studies.

The effect of data transfer optimization in a heterogeneous satellite-terrestrial network on the lifetime of battery-powered devices is another important issue. Normally, the question is raised in relation to end devices running LoRa, IEEE 802.15.4 and similar energy-efficient protocol stacks, while network gateways are considered to be supplied with a constant energy source. With the active development of micro-energy harvesters, more complicated and resource-intensive network devices can become fully autonomous as well.

Finally, network clustering can be additionally applied to divide a complex data acquisition network into multiple components, each comprising a satellite gateway. Within each component, incoming data streams can be aligned in such a way that the GDEP container utilization rate is maximized.

## Figures and Tables

**Table 1:** Comparison of data presentation formats.

Format	Requires Predefined Data Structure	Has Embedded Compression	Allows Binary Data Transmission	Use Data Optimization Techniques
JSON	No	No	No	No
BSON	No	No	Yes	No
CBOR	No	No	Yes	Yes
MsgPack	No	No	Yes	Yes
JSONC	No	Yes	Yes	Yes
Protobuf	Yes	No	Yes	Yes

**Table 2:** Comparison of data transfer encoded in JSON and in the proposed format (Protobuf).

Number of Sensors	Total Number of Messages	Average Message Size in JSON Format (bytes)	Average Message Size in the Protobuf Format (bytes)	Message Size Ratio (3/4)
1	32	340	78	4.36
2	90	369	82	4.5
3	153	398	86	4.62
4	170	427	90	4.74
5	177	456	94	4.85
6	143	485	98	4.94
7	107	514	102	5.04
8	54	542	106	5.12
9	36	572	110	5.2
10	21	601	114	5.27
11	5	629	118	5.33
12	3	664	122	5.44
13	2	694	126	5.5
Total	1000	454	94	4.82

**Table 3:** Comparison of packaging algorithms.

Criteria	Algorithm 1	Algorithm 2	Algorithm 3
Number of sent messages	1000	1000	1000
Number of used containers	334	333	330
Average number of messages per container	2.99	3	3.03
Average container size in bytes	280.99	281.83	284.4
Container utilization, %	87.8	88	88.8
Number of iterations for packing messages into containers	1000	167,347	667,518
The delay associated with the process of packing messages into containers in seconds	3 × T	1000 × T	1000 × T
